# Association of Circulating Neprilysin with BMI, Cardiovascular Health, and Kidney Function in High-Risk Pregnancies: A Pilot Study

**DOI:** 10.3390/biomedicines13010052

**Published:** 2024-12-28

**Authors:** Kaltrina Kutllovci Hasani, Azra Kulovic-Sissawo, Adam Saloň, Christina Stern, Karoline Mayer-Pickel, Mila Cervar-Zivkovic, Nandu Goswami, Herbert Fluhr, Ursula Hiden

**Affiliations:** 1 Department of Obstetrics and Gynaecology, Medical University of Graz, Auenbruggerplatz 14, 8036 Graz, Austriachristina.stern@medunigraz.at (C.S.); mila.cervarzivkovic@medunigraz.at (M.C.-Z.); ursula.hiden@medunigraz.at (U.H.); 2 Vascular Biology Centre, Augusta University, 1460 Laney Walker Blvd, Augusta, GA 30912, USA; 3 Gravitational Physiology and Medicine Research Unit, Division of Physiology and Pathophysiology, Otto Löwi Research Centre of Vascular Biology, Immunity and Inflammation, Medical University of Graz, Neue Stiftingtalstrasse 6, 8010 Graz, Austria; 4 Centre for Space and Aviation Health, Mohammed Bin Rashid University of Medicine and Health Sciences, Al Razi St-Umm Hurair 2, Dubai 505055, United Arab Emirates

**Keywords:** soluble neprilysin, vascular health, high-risk pregnancies

## Abstract

Background/Objectives: Inadequate cardiovascular adaptation during pregnancy impairs endothelial function and vascular resistance, contributing to complications such as pre-eclampsia (PE) and gestational hypertension (GH). Neprilysin (NEP), a protease involved in vascular regulation, has been linked to PE, but its role in endothelial function and vascular adaptation remains unclear. This pilot study investigates the associations between soluble neprilysin (sNEP) and markers of vascular and renal function in high-risk pregnancies without PE. Methods: Observed parameters were analyzed in 29 high-risk pregnant women across early, mid-, and late pregnancy. sNEP levels were analyzed alongside body mass index (BMI), endothelial dysfunction (ADMA), arterial stiffness (pulse wave velocity, PWV), retinal microvasculature (central retinal arteriolar and venular equivalents, CRAE and CRVE), and kidney function markers. The impact of gestational hypertension (GH) and prior smoking on sNEP levels was also evaluated. Results: In early and mid-pregnancy, sNEP was inversely associated with BMI. During mid-pregnancy, sNEP showed a positive correlation with CRAE and an inverse correlation with PWV, suggesting reduced arterial stiffness. By late pregnancy, sNEP was positively associated with glomerular filtration rate and inversely correlated with creatinine and protein levels, reflecting improved kidney function. Women with GH exhibited elevated sNEP, while former smokers had lower sNEP levels in early pregnancy. Conclusions: These findings suggest that sNEP plays a role in vascular and renal adaption during pregnancy, offering new perspectives on vascular tone regulation in high-risk pregnancies. Further research is needed to clarify these mechanisms and their clinical relevance.

## 1. Background

Inadequate cardiovascular adaptation during pregnancy impacts endothelial function and vascular resistance, which can lead to complications such as pre-eclampsia (PE) and gestational hypertension (GH) [1]. PE, a multisystem disorder, affects 2–8% of pregnancies globally and is a significant contributor to maternal mortality [2]. It is characterized by the onset of hypertension after 20 weeks of gestation, accompanied by one or more new onset conditions such as proteinuria, maternal organ dysfunction, or utero-placental insufficiency [1,3]. GH is defined as de novo blood pressure elevations (greater than 140/90 mm Hg) occurring after 20 weeks gestation without associated organ system dysfunction [3]. GH and PE share similar risk factors, including obesity, parity, and a history of previous pre-eclamptic pregnancies [4,5,6]. Screening methods for GH focus primarily on monitoring blood pressure, especially in women with known risk factors [3]. Otherwise, the screening process combines maternal risk factors with mean arterial pressure (MAP), serum placental growth factor (PlGF), pregnancy-associated plasma protein A (PAPP-A), and uterine artery pulsatility index (UTPI) to identify women at high risk of developing preeclampsia (PE) [7]. Prophylactic low-dose aspirin is recommended for these women before 16 weeks gestation to reduce the risk of PE, as early intervention can improve maternal outcomes [8,9].

Due to the complexity of hypertensive disorders in pregnancy, it is important to understand the involvement of additional factors that may be involved in disease development and may serve as novel biomarkers is crucial. Indeed, the soluble, circulating form of the protein neprilysin (NEP) has been suggested as a potential candidate soluble [10]. NEP, a membrane-bound metallopeptidase, plays a pivotal role in deactivating signaling peptides that regulate critical physiological functions, including cardiovascular function, kidney function, metabolism, and immune responses [11]. Specifically, it degrades vasoactive peptides, including vasodilators (e.g., atrial natriuretic peptide, bradykinin) and vasoconstrictors (e.g., endothelin-1, angiotensin II). NEP is present in various organs such as the kidney, brain, heart, adrenal glands, lungs, gastrointestinal tract, and thyroid, highlighting its extensive physiological impact [12,13,14,15]. The soluble form of neprilysin (sNEP) is released into the blood, urine, and cerebrospinal fluid, with endothelial cells contributing to the overall NEP pool [11,12,15,16]. sNEP reflects systemic NEP activity and plays a key role in blood pressure regulation [17] and has been associated with cardiovascular health in various populations [18,19]. sNEP maintains vascular tone and fluid balance—functions that are particularly critical during pregnancy to support maternal adaptation and fetal growth. Furthermore, sNEP is produced by the placenta, and placenta-derived extracellular vesicles carry NEP into the maternal circulation, where it may regulate local and systemic vascular responses to accommodate the growing fetal demands [20]. In PE, elevated NEP in these placenta-derived extracellular vesicles suggests a dysregulation of NEP transport to the mother, contributing to impaired vascular adaptation and hypertension [20]. These findings highlight the need to explore the role of sNEP as both a potential biomarker and a regulatory factor in high-risk pregnancies.

However, there is limited literature on sNEP and its impact on cardiovascular and renal function in pregnancy complications. In patients with heart failure, elevated sNEP levels are associated with an increased risk of cardiovascular events [18], whereas an adverse cardiometabolic and smoking profile in the general population over the age of 45 is associated with reduced sNEP levels [19].

The objective of this pilot study was to evaluate the association between sNEP and key cardiovascular markers, including endothelial function, hemodynamics, arterial stiffness, retinal microvascular profile, and renal function during pregnancy. In addition, the concentration of sNEP in the GH group and the effect of previous smoking were assessed. By focusing on a cohort of high-risk pregnant women predisposed to PE, we aimed to elucidate the role of NEP in cardiovascular adaptation mechanisms during pregnancy.

## 2. Materials and Methods

### 2.1. Study Population and Design

The investigations for this study were conducted from November 2019 to September 2022 at the Department of Obstetrics and Gynecology, Medical University of Graz. The study protocol was approved by the Ethics Committee of the Medical University of Graz (EK: 31-541 ex 18/19). All procedures adhered to high clinical standards and complied with the latest version of the Declaration of Helsinki. Informed written consent was obtained from all participants after providing detailed information about the study. The study has been registered on ClinicalTrials.gov (NCT06645340).

As this is a pilot study, the sample size was determined based on the maximum feasibility of participant recruitment during the study period. This pilot study examined the association between sNEP concentrations and cardiovascular parameters, including endothelial function, arterial stiffness, retinal microvascular changes, and renal biochemical function markers, in high-risk pregnancies. Differences in sNEP concentrations were explored between normotensive high-risk women (control group) and those who develop gestational hypertension. Additionally, the study investigated sNEP levels between previous smokers and non-smokers within the control group. This preliminary analysis aimed to provide valuable insights into the relationship between sNEP and key cardiovascular parameters, sNEP concentrations in GH, and the impact of previous smoking on sNEP levels, serving as a basis for larger future studies.

#### 2.1.1. Participants

Participants were pregnant women carrying a single fetus, assessed at the Medical University of Graz as being at high risk of developing PE as determined by first-trimester PE screening [8] between gestational weeks 11 + 0 and 13 + 6. Participants were included in the study if they had a positive PE screening, a history of the condition, were taking 150 mg of aspirin daily, and were over 18 years of age. Women were excluded if they had multiple pregnancies, fetal anomalies, chronic kidney disease, or had discontinued aspirin use.

#### 2.1.2. Study Protocol

**Figure 1:**
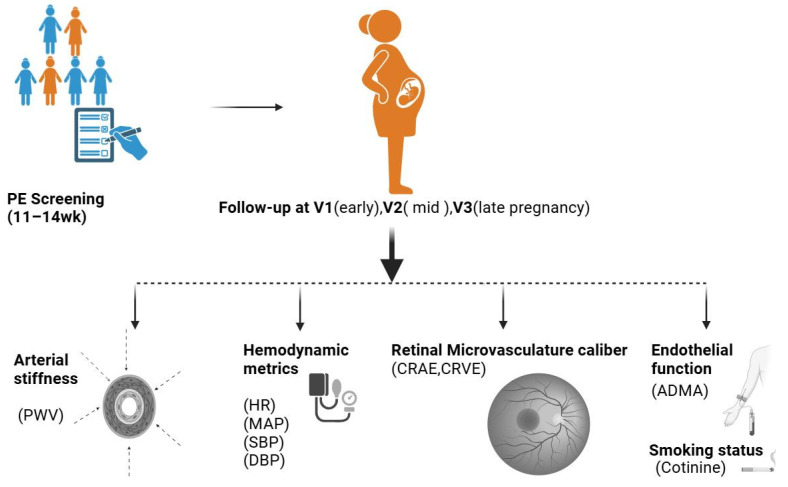
Simplified overview of the study workflow. The initial phase of the study involved selection of eligible participants through pre-eclampsia (PE) screening and familiarization with the study protocol. The study consisted of three visits: V1 (early pregnancy, 11–16 weeks), V2 (mid-pregnancy, 24–28 weeks), and V3 (late pregnancy, 34–37 weeks). At all visits, data were collected on arterial stiffness, retinal microvascular profiles, endothelial biomarkers, and biochemical parameters for renal function, as well as serum concentrations of ADMA and sNEP concentration. The illustration was generated using BioRender (www.biorender.com). Abbreviations: sNEP, soluble neprilysin; SBP, systolic blood pressure; DBP, diastolic blood pressure; MAP, mean arterial pressure; PWV, pulse wave velocity; HR, heart rate; CRAE, central retinal arteriolar equivalent; CRVE, central retinal venular equivalent; ADMA, asymmetric dimethylarginine.

Qualified participants were instructed to abstain from exercise and other stimulating activities for 48 h prior to testing. Measurements were conducted in three phases: first visit (V1): early pregnancy (11–16 weeks), second visit (V2): mid-pregnancy (24–28 weeks), and third visit (V3): late pregnancy (34–37 weeks). An overview of the study workflow is presented in Figure 1.

#### 2.1.3. PE Screening

Screening for PE was conducted according to the recommendations of the Fetal Medicine Foundation (FMF) [8]. This screening integrates maternal risk factors and biophysical markers measured between 11 + 0 and 13 + 6 weeks of gestation. Maternal risk factors assessed included age, weight, height, and medical history with particular emphasis on pre-existing conditions such as chronic hypertension, diabetes, autoimmune disorders, and previous incidence of PE [8,21]. Blood pressure was measured, and the mean arterial pressure (MAP) was calculated. Biochemical markers, including Pregnancy-Associated Plasma Protein-A (PAPP-A) and Placental Growth Factor (PlGF), were quantified. Additionally, uterine artery Doppler studies were performed to determine the Pulsatility Index (PI), which provides an assessment of uterine artery blood flow resistance. These parameters were used to estimate high-risk women [8]. Women identified as high risk were prescribed 150 mg of aspirin daily until 36 weeks of gestation [9].

#### 2.1.4. Diagnostics Criteria for GH

GH was categorized by systolic blood pressure readings of 140 mmHg or higher, or diastolic blood pressure readings of 90 mmHg or higher, recorded twice with a 4-hour interval after 20 weeks gestation in women with previously normal blood pressure without accompanying proteinuria or signs of systemic disorders [3].

#### 2.1.5. Arterial Stiffness

Arterial stiffness was estimated by pulse wave velocity (PWV) using the VICORDER^®^ (SMT Medical GmbH & Co. KG, Würzburg, Germany). Oscillo metric pressure cuffs were placed at two sites: the carotid artery and the upper part of the right thigh. Participants rested in a supine position with the upper body elevated 30° for 10 min prior to measurement. The distance between the carotid and femoral arteries was measured from the suprasternal notch to the midpoint of the cuff on the right thigh. The pressure waveforms at the carotid artery and right thigh were recorded simultaneously, and the PWV, which refers to the speed at which the pressure wave generated by the contraction of the heart travels along the arterial tree, was calculated in meters per second (m/s) using the VICORDER^®^ software, version 2007 (Skidmore Medical). 

#### 2.1.6. Hemodynamic Parameters

Pulse wave velocity was assessed by averaging blood pressure and heart rate from two separate measurements. Mean arterial pressure (MAP) was then calculated from the diastolic and systolic blood pressures (DBP, SBP) using the formula MAP = DBP + (SBP − DBP)/3.

#### 2.1.7. Retinal Microvasculature Caliber

Optic disc-centered images of both eyes were captured using a non-mydriatic, hand-held, 30° field-of-view digital retinal camera (Optomed Aurora, Optomed Oy, Oulu, Finland) and a resolution of 1536 × 1536. To ensure data accuracy, retinal vessel measurements were performed by a single trained grader blinded to participant details using the MONA REVA software, version 2.1.1 developed at VITO (Boeretang, Belgium) [22]. The grader was specifically trained by MONA REVA staff to adhere to standardized vessel segmentation and classification protocols, ensuring consistency and reliability of measurements. As all measurements were conducted by a single grader, inter-rater reliability was not applicable. The software’s algorithm automatically segmented the retinal vessels, and post-processing techniques such as double thresholding, blob extraction, elimination of small connected regions, and hole filling were applied. Diameters of the retinal arterioles and venules, located 0.5 to 1 disc diameter from the optic disc margin, were measured automatically. The grader also verified and adjusted vessel measurements and classifications using the MONA REVA vessel editing toolbox [22]. The Central Retinal Artery Equivalent (CRAE) and Central Retinal Venular Equivalent (CRVE) were calculated in micrometers (µm), based on the diameters of the six largest arterioles and venules using the revised Parr–Hubbard formula [23].

#### 2.1.8. sNEP, ADMA, and Cotinine Measurements in Serum

Blood samples were centrifuged at 800 rpm for 10 min without anticoagulants and stored as serum samples at −80 °C. The concentrations of sNEP, ADMA, and cotinine were analyzed using enzyme-linked immunosorbent assay (ELISA) kits. The circulating concentration of sNEP was measured using the Human Neprilysin DuoSet ELISA (R&D Systems, Minneapolis, MN, USA). ADMA is recognized as an indirect biomarker of endothelial function [24], and serum ADMA levels were measured using an ELISA kit (Immunodiagnostic AG, Bensheim, Germany). Serum cotinine levels were quantified using an Abnova ELISA kit (KA0930, Taipei, Taiwan) following the manufacturer’s recommended protocol. Serum cotinine levels of active smokers are above 100 ng/mL, whereas non-smokers typically have levels between 0 and 5 ng/mL.

#### 2.1.9. Blood Parameters for Renal Function

Data on renal functions were collected exclusively during routine visits or scheduled study appointments using laboratory measurements from the Clinical Institute for Medical and Chemical Laboratory Diagnostics at the Medical University of Graz. Data were collected for the following parameters: creatinine (mg/dL); estimated glomerular filtration rate (eGFR, mL/min); total protein (g/dL); and albumin (g/dL).

### 2.2. Statistical Analysis

Comprehensive plausibility checks were performed to identify and manage outliers or influential points within our dataset. The concentration values of sNEP were logarithmically transformed. The normal distribution of data was tested using Shapiro–Wilk tests (with *p* > 0.05 indicating a normal distribution) and visually assessed using Q–Q plots. Pearson and Spearman correlation analyses were used to evaluate the relationships between variables, depending on the normality of distribution. For variables meeting the normality criteria, group means were compared across the three stages of pregnancy (V1, V2, V3) using independent samples *t*-tests. For variables that did not show a normal distribution, the Mann–Whitney U test was employed to compare group medians at the same stages of pregnancy. Since this is a pilot study, the number of participants varies across visits. Missing data were handled by performing analyses on available cases without specific imputation procedures. The sample size for each visit is reported in the corresponding tables to maintain transparency regarding the number of observations included in each analysis.

Variation in the number of participants between the visits was due to loss of follow-up, unavailability of participants, incomplete data collection, or undetectable sNEP concentration in certain samples. To minimize bias, analyses were performed on available cases without imputation procedures. Although this limitation affects statistical power, it is a recognized constraint in pilot studies and provides valuable insights for future research.

All statistical tests were two-sided, with a significance threshold of *p* < 0.05. Ninety-five percent confidence intervals were reported for all estimates. Statistical analyses were performed with SPSS version 27.0. GraphPad Prism version 9 was used to generate graphs.

## 3. Results

Of the 110 women initially screened for PE, 43 were identified as having high-risk pregnancies and met the inclusion criteria. Of these participants, 29 did not develop PE and were included in the analysis to evaluate the association between sNEP and cardiovascular and renal biochemical parameters. Of these high-risk participants, six developed GH during pregnancy, while the remaining 23 had normotensive pregnancies until delivery and were identified as the control group. The mean BMI of the participants remained within the normal range, with 13 being former smokers who had quit after a positive pregnancy test, and the remainder being non-smokers. Detailed participant characteristics, including demographic data, are shown in Table 1.

### 3.1. Correlations of sNEP with Anthropometrics, Retinal Microvasculature Profile, Vascular Biomarkers, and Renal Biochemical Parameters

**Figure 2:**
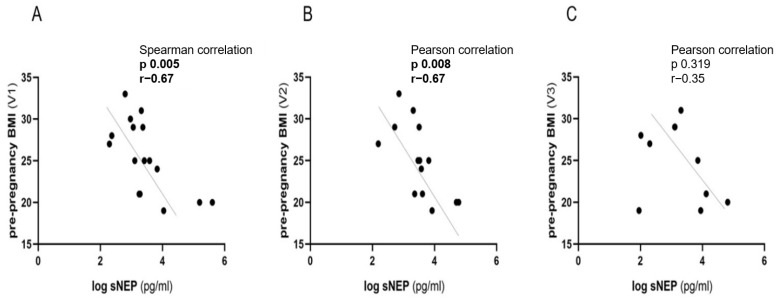
Correlation of sNEP and maternal pre-pregnancy BMI in control group of high-risk pregnancies in early (V1), mid- (V2), and late (V3) pregnancy. (**A**) Correlation of sNEP with BMI at V1, (**B**) correlation of sNEP with BMI at V2, (**C**) correlation of sNEP with BMI at V3; sNEP concentration values were log-transformed. Pearson correlation was used for normally distributed data, and Spearman correlation was used for non-normally distributed data. The results were considered statistically significant and indicative of a correlation when *p* < 0.05. sNEP: soluble neprilysin; BMI: body mass index.

The correlations of sNEP with BMI, age, retinal microvascular profiles, endothelial and arterial stiffness biomarkers, as well as biochemical parameters related to renal function, were analyzed in the normotensive control group during early (V1), mid- (V2), and late (V3) pregnancy. Descriptive data for the control group (n = 23), including all parameters observed at the three stages of pregnancy, are presented in Table 2. sNEP demonstrated a negative correlation with maternal pre-pregnancy BMI during early and mid-pregnancy. Correlation coefficients were recorded during early pregnancy (V1) (r = −0.67; *p* = 0.005) and mid-pregnancy (V2) (r = −0.67; *p* = 0.008), indicating a strong negative relationship. However, at late pregnancy (V3), the correlation coefficient decreased to (r = −0.35; *p* = 0.319), which did not reach statistical significance (Figure 2A–C, Table 3). sNEP did not exhibit a statistically significant correlation with other observed biomarkers and laboratory parameters at V1, as detailed in Table 3.

**Figure 3:**
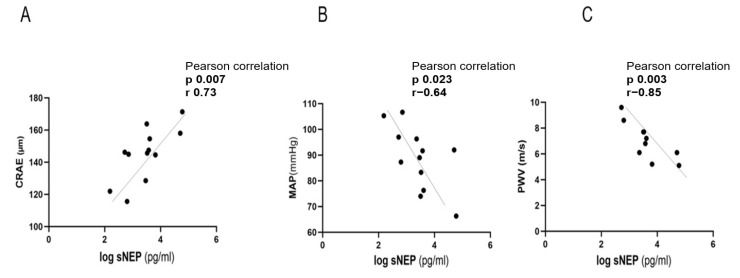
Correlation of sNEP with CRAE, MAP, and PWV in control group of high-risk pregnancies at mid-pregnancy (V2). sNEP concentration values were log-transformed. (**A**) Correlation of sNEP with CRAE, (**B**) correlation of sNEP with MAP, (**C**) correlation of sNEP with PWV. sNEP: soluble neprilysin; CRAE: central retinal artery equivalent; MAP: mean arterial pressure; PWV: pulse wave velocity.

At mid-pregnancy (V2), sNEP demonstrated a positive correlation with the central retinal artery equivalent (CRAE), possessing a correlation coefficient of (r = 0.73; *p* = 0.007) (Figure 3A). However, there was no significant correlation between sNEP and the central retinal vein equivalent (CRVE) (Table 3). Additionally, sNEP showed a negative correlation with mean arterial pressure (MAP), with a coefficient of (r = −0.64; *p* = 0.023) (Figure 3B), and a strong negative correlation with pulse wave velocity (PWV), with a coefficient of (r = −0.85; *p* = 0.003) (Figure 3C). No significant correlations were observed between sNEP and other parameters at V2 (Table 3).

**Figure 4:**
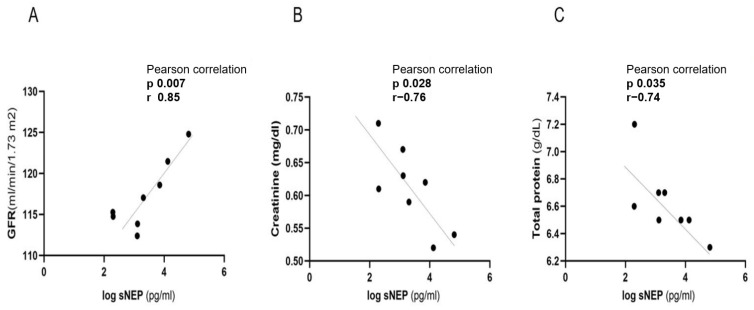
Correlation of sNEP with GFR, creatinine, and total protein in a healthy control group of high-risk pregnancies at late pregnancy (V3). sNEP concentration values were log-transformed. (**A**) Correlation of sNEP with GFR, (**B**) correlation of sNEP with Creatinine, (**C**) correlation of sNEP with total protein. sNEP: soluble neprilysin; GFR: glomerular filtration rate.

In late pregnancy (V3), a strong positive correlation was observed between sNEP and glomerular filtration rate (GFR) (r = 0.85, *p* = 0.007; Figure 4A). At the same pregnancy stage, sNEP also exhibited negative correlations with creatinine (r = −0.76, *p* = 0.028; Figure 4B) and total protein levels (r = −0.74, *p* = 0.035; Figure 4C). Additionally, sNEP did not exhibit a statistically significant correlation with other observed biomarkers and laboratory parameters at V3, as detailed in Table 3.

### 3.2. Comparison of sNEP Levels Between Normotensive Controls and GH Group

**Figure 5:**
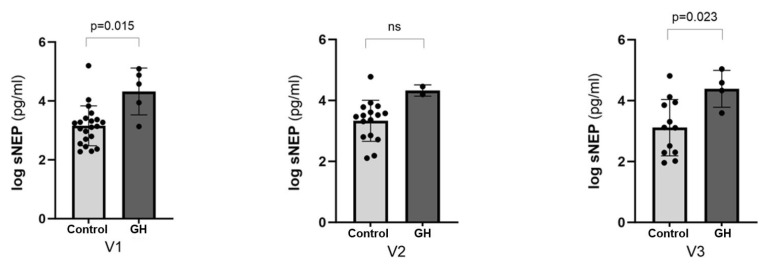
Comparison of sNEP concentrations between control high-risk and GH group. Pregnancy stage: early pregnancy (visit 1, V1), mid-pregnancy (visit 2, V2), and late pregnancy (visit 3, V3). sNEP concentration values were log-transformed. Data are presented as means ± SD, and group means were compared using *t*-tests. Statistical significance was considered at *p* < 0.05. “ns” stands for “not significant”, indicating that the observed comparisons did not achieve statistical significance (*p* > 0.05). Control group (n = 23) and GH group (n = 6), represented as points in the graph; GH: gestational hypertension; sNEP: soluble neprilysin.

Comparison of sNEP concentrations between the normotensive control group and the GH group revealed a significant increase in the GH group at V1 (*p* = 0.015) and V3 (*p* = 0.023), with a trend towards significance at V2 (*p* = 0.059) (Figure 5).

### 3.3. Comparison of sNEP Levels Between Previous Smokers and Non-Smokers

When analyzing the effect of smoking history on sNEP concentration in the normotensive control group, we observed a decrease in sNEP in previous smokers (women who quit smoking after a positive pregnancy test) compared to non-smokers in early pregnancy (V1, *p* = 0.022). The difference declined and lost significance in mid- (V2) and late pregnancy (V3) (Table 4). Measurement of serum cotinine levels in these women confirmed their non-smoking status at the time of measurement during the study.

## 4. Discussion

This pilot study investigated the role of sNEP in cardiovascular adaptation and renal function in high-risk pregnancies and revealed important associations with maternal BMI, vascular and renal function, gestational hypertension, and smoking history. These findings provide novel insights into the clinical relevance of sNEP and its potential impact on maternal adaptation to pregnancy.

The inverse correlation between early and mid-pregnancy sNEP levels with pre-pregnancy BMI highlights the influence of maternal weight on sNEP regulation. Women with higher pre-pregnancy BMI had lower sNEP levels, which is consistent with previous findings that maternal overweight is associated with the downregulation of umbilical cord blood NEP levels [16]. Conversely, a non-pregnant cohort of 135 males and females between 18 and 70 years of age shows a positive correlation between sNEP and BMI [25], suggesting different regulatory mechanisms during pregnancy. This discrepancy may result from metabolic [21] or inflammatory processes specific to pregnancy that alter sNEP expression. Pregnancy is characterized by elevated levels of hormones, cytokines, and growth factors, many of which follow a temporal or concentration-based progression throughout gestation [26,27]. Notably, neprilysin expression is influenced by factors associated with the hormonal, metabolic, and pro-inflammatory conditions of pregnancy. Estrogen [28] and pro-inflammatory cytokines [29] upregulate NEP expression, whereas leptin downregulates it [30]. Interestingly, no correlation was found between sNEP and BMI in pre-eclamptic patients [31], further emphasizing the complexity of this relationship. These findings underscore the importance of pre-conception interventions targeting BMI to potentially modulate sNEP levels and thereby improve vascular and renal function during pregnancy.

The observed associations between sNEP levels and vascular function in mid-pregnancy support its protective role. Higher sNEP levels were positively correlated with retinal arteriolar caliber (CRAE) and inversely correlated with arterial stiffness (PWV) and mean arterial pressure (MAP). These findings suggest that sNEP may promote vasodilation and vascular health through its role in metabolizing vasoactive peptides such as angiotensin II and endothelin-1 [17,19]. Increased retinal vasodilation, reflected by wider CRAE, is consistent with reports of elevated NEP expression in the placenta and peripheral circulation during pregnancy [2,5,32]. Similar benefits of NEP have been observed in non-pregnant populations treated with angiotensin receptor-neprilysin inhibitors (ARNi), which reduce arterial stiffness and MAP by decreasing PWV [33,34]. However, the lack of correlation between sNEP and ADMA, a marker of endothelial dysfunction [24,35], suggests that the role of sNEP in vascular health during pregnancy may involve mechanisms other than endothelial function.

Renal function parameters in late pregnancy were also linked to sNEP levels, with higher sNEP levels being associated with improved glomerular filtration rate (GFR) and lower creatinine and total protein levels. These findings highlight the potential role of sNEP in optimizing renal blood flow and filtration efficiency, probably by degrading peptides such as natriuretic peptides, angiotensin II, and bradykinin [12,13]. In contrast to the general population, where no association between sNEP and GFR has been observed [19], these results suggest that pregnancy-specific demands modulate sNEP activity to support renal function. Notably, elevated sNEP levels in PE have been associated with increased creatinine and disease severity [31,36], further differentiating its role in normotensive and hypertensive pregnancies.

Elevated sNEP levels in women with GH may indicate either a compensatory mechanism to alleviate hypertension or a dysregulation contributing to vascular complications. Similar increases in sNEP have been reported in PE, where they correlate with disease severity [20,31,36]. Increased placental NEP expression and its role in the degradation of vasoactive peptides may explain this upregulation in hypertensive pregnancies [18,32]. Whether these elevated levels represent an adaptive or maladaptive response requires further investigation.

Smoking status was validated by participant self-reporting and confirmed by serum cotinine measurements, which showed concentrations below the threshold for passive smoking exposure. This ensured that participants classified as non-smokers or previous smokers were not exposed to passive smoking during the study, allowing us to interpret the observed parameters without the confounding effects of smoking. Interestingly, smoking history influenced sNEP levels, with former smokers who quit smoking when pregnancy was confirmed having lower sNEP levels in early pregnancy compared with non-smokers. This observation is consistent with findings in the general population, where smoking is associated with lower NEP levels [19]. While sNEP levels in former smokers showed no statistical difference compared to non-smokers in mid- and late pregnancy, the data in Table 4 indicate that this lack of significance does not reflect full normalization in former smokers. Instead, the nearly identical sNEP levels observed in former smokers during early and late pregnancy suggest that the impact of smoking history on sNEP regulation persists throughout pregnancy. The reduction in sNEP levels among non-smokers during late pregnancy may partially account for the diminishing statistical differences, rather than indicating recovery in sNEP regulation among former smokers. The observed decrease in sNEP levels in non-smokers during late pregnancy merits further investigation, as it may reflect pregnancy-specific physiological adaptations, including vascular remodeling, hormonal shifts, or metabolic demands, which could modulate NEP activity independently of smoking history. Exploring these broader mechanisms in future research is essential to better understand the interaction between smoking history and pregnancy-related vascular changes. These findings underscore the lasting impact of preconception smoking on maternal vascular markers and highlight the need for longitudinal studies to explore the mechanisms driving these changes and their implications for maternal and fetal health.

As a pilot study, these findings provide a basis for future research, but the results should be interpreted with caution due to the limited sample size, which is a major limitation of the study. Moreover, missing data were analyzed as available cases without specific imputation procedures. Although this approach may introduce bias, it is consistent with the exploratory nature of the study, which aims to generate hypotheses for future adequately powered investigations. The COVID-19 pandemic disrupted follow-up visits, resulting in incomplete longitudinal data. Additionally, the lack of sNEP activity measurements limits understanding of its functional role. We recognize that unmeasured confounders, such as dietary habits, socioeconomic status, and medication compliance, may have influenced the cardiovascular and renal parameters observed in this study. While standardized protocols were followed to minimize variability, future studies should include these variables to provide a more comprehensive understanding of their impact on sNEP levels and related outcomes.

## 5. Conclusions

This pilot study highlights the significant impact of sNEP on vascular and renal function in high-risk pregnancies. Lower sNEP levels were associated with higher pre-pregnancy BMI, suggesting that maternal overweight may downregulate sNEP and impair physiological adaptation, underscoring the role of obesity as a risk factor for hypertensive disorders in pregnancy. Conversely, higher sNEP levels were associated with beneficial outcomes, including reduced arterial stiffness, wider retinal arteriolar calibers (CRAE), improved glomerular filtration rate (GFR), and lower creatinine and total protein levels. These findings may indicate a protective role of sNEP in pregnancy, supporting vascular health and renal function.

The elevated sNEP levels observed in GH may represent a compensatory mechanism to counteract vascular strain or a dysregulated response contributing to hypertensive complications. The observed reduction in sNEP levels among prior smokers highlights the influence of preconception lifestyle factors on maternal adaptation to pregnancy.

As a pilot study, these findings underscore the need for further research to elucidate the mechanisms by which sNEP exerts its effects, to explore its interactions with vasoactive peptides, and to evaluate its therapeutic potential in mitigating complications during high-risk pregnancies. Addressing current limitations and expanding the scope of research will improve our understanding of the role of sNEP and inform targeted strategies to improve maternal and fetal health outcomes.

## Figures and Tables

**Table 1:** Characteristics of study participants.

Characteristics	
Total high-risk participants (n)	29
Age (years)	34.1 ± 4.1
Pre-pregnancy BMI (kg/m²)	25.7 ± 5.3
Ethnicity white/black (n)	28/1
Previous smokers: n (%)	13 (45%)
Nulliparous: n (%)/multiparous: n (%)	11 (38%)/18 (62%)
Spontaneous conception/IVF (n)	27/2
Outcome with GH: n (%)	6 (20.7%)

Data are presented as means ± SD, or as total numbers (n) and percentages (%) for categorical variables. BMI: body mass index; GH: gestational hypertension; IVF: In vitro fertilization.

**Table 2:** Descriptive data of sNEP levels and parameters for endothelial function, arterial stiffness, retinal microvasculature, and renal function in the control group (n = 23) at early (V1), mid- (V2), and late (V2) pregnancy stages.

Stage of Pregnancy	V1 Mean/Median ± SD/IQR/n	V2 Mean/Median ± SD/IQR/n	V3 Mean/Median ± SD/IQR/n
sNEP (pg/mL)	1867 ± 3218/19	3267 ± 5337/16	1291 ± 8170/12
SBP (mmHg)	122.5 ± 15.4/16	123.2 ± 15.2/13	129.6 ± 22.4/8
DBP (mmHg)	68.7 ± 9.5/16	70.0 ± 11.2/13	78.7 ± 15.7/8
MAP (mmHg)	87.4 ± 11.6/16	87.7 ± 12.2/13	95.7 ± 17.1/8
HR (beats/min)	77.4 ± 10.9/16	77.7 ± 6.9/13	88.3 ± 15.5/8
PWV (m/s)	7.2 ± 1.1/16	7.01 ± 1.4/10	7.7 ± 1.2/5
CRAE (µm)	145.2 ± 15.5/17	146.4 ± 16.2/13	143.4 ± 14.8/8
CRVE (µm)	221.8 ± 18.6/17	226 ± 22.4/13	223.9 ± 20.9/8
ADMA (µmol/L)	0.45 ± 0.0/22	0.44 ± 0.0/17	0.48 ± 0.0/12
Creatinine (mg/dL)	0.5 ± 0.0/21	0.5 ± 0.0/16	0.6 ± 0.0/11
eGFR (mL/min)	118.7 ± 8.6/21	116.9 ± 5.8/16	117.7 ± 4.4/11
Total protein (g/dL)	7.0 ± 0.3/21	6.7 ± 0.2/16	6.5 ± 0.3/11
Albumin (g/dL)	4.0 ± 0.1/21	3.7 ± 0.1/16	3.5 ± 0.1/11

Data are presented as means ± SD for all normally distributed parameters, while not normally distributed data are presented as median ± IQR, (n) indicates the total number of participants for each parameter measured at the corresponding pregnancy visits. V1: visit 1 in early pregnancy; V2: visit 2 in mid-pregnancy; V3: visit 3 in late pregnancy. sNEP: soluble neprilysin; SBP: systolic blood pressure; DBP: diastolic blood pressure; MAP: mean arterial pressure; PWV: pulse wave velocity; HR: heart rate; CRAE: central retinal arteriolar equivalent; CRVE: central retinal venular equivalent; ADMA: asymmetric dimethylarginine; eGFR: estimated glomerular filtration rate.

**Table 3:** Correlation between sNEP concentrations and retinal microvascular caliber, endothelial function, hemodynamic biomarkers, and kidney biochemical parameters in the control group (n = 23).

	sNEP V1		sNEP V2		sNEP V3	
	r	*p*-Value	r	*p*-Value	r	*p*-Value
Age (years)	−0.277	0.281	−0.191	0.496	−0.389	0.236
Pre-pregnancy-BMI (kg/m^2^)	**−0.670**	**0.005**	**−0.670**	**0.008**	−0.35	0.319
SBP (mmHg)	−0.198	0.670	**−0.675**	**0.016**	−0.140	0.791
DBP (mmHg)	−0.270	0.558	**−0.598**	**0.040**	0.367	0.474
MAP (mmHg)	−0.179	0.713	**−0.648**	**0.023**	0.144	0.785
HR (beats/min)	0.400	0.517	−0.324	0.331	−0.0002	0.999
PWV (m/s)	−0.400	0.750	**−0.854**	**0.003**	0.677	0.209
CRAE (µm)	0.514	0.052	**0.731**	**0.007**	0.269	0.559
CRVE (µm)	0.239	0.389	0.334	0.288	0.709	0.074
ADMA (µmol/L)	0.210	0.387	0.360	0.171	0.067	0.836
Creatinine (mg/dL)	0.153	0.556	−0.271	0.349	**−0.762**	**0.028**
eGFR (mL/min)	0.025	0.928	0.399	0.158	**0.851**	**0.007**
Total protein (g/dL)	0.154	0.566	−0.433	0.122	−**0.741**	**0.035**
Albumin (g/dL)	0.219	0.411	−0.297	0.302	−0.026	0.950

sNEP concentration values were log-transformed. To evaluate the relationships between variables, Pearson and Spearman correlation analyses were employed depending on the normality of distribution. Data are presented with correlation coefficients (r) and *p*-values. Values highlighted with bold text indicate measurements that achieved statistical significance (*p* < 0.05). sNEP: soluble neprilysin; SBP: systolic blood pressure; DBP: diastolic blood pressure; MAP: mean arterial pressure; PWV: pulse wave velocity; HR: heart rate; CRAE: central retinal arteriolar equivalent; CRVE: central retinal venular equivalent; ADMA: asymmetric dimethylarginine; eGFR: estimated glomerular filtration rate.

**Table 4:** Comparison of sNEP levels between previous smokers and non-smokers.

	Stage	Previous Smokers (n = 9)	Non-Smokers (n = 14)	*p* Value
log sNEP	V1	2.82 ± 0.52	3.61 ± 0.92	0.022
log sNEP	V2	3.20 ± 0.68	3.59 ± 0.66	0.337
log sNEP	V3	2.88 ± 0.94	3.22 ± 0.95	0.581

sNEP concentration values were log-transformed. Data are presented as means ± SD. Values highlighted with bold text indicate measurements that achieved statistical significance (*p* < 0.05). Independent *t*-tests were used. Statistical significance is considered at *p* < 0.05. V1: visit 1, early pregnancy; V2: visit 2, mid-pregnancy; V3: visit 3, late pregnancy; sNEP: soluble neprilysin.

## Data Availability

The datasets generated from the current study are available from the corresponding author on request.
